# Generation of mode-locked states of conventional solitons and bright-dark solitons in graphene mode-locked fiber laser

**DOI:** 10.1007/s12200-023-00067-2

**Published:** 2023-06-02

**Authors:** Zixiong Li, Mingyu Li, Xinyi Hou, Lei Du, Lin Xiao, Tianshu Wang, Wanzhuo Ma

**Affiliations:** Department of Optical Engineering, School of Opto-Electronic Engineering, Changchun University of Science and Technology, Changchun, 130022 China

**Keywords:** Fiber laser, Graphene, Conventional soliton, Bright-dark soliton pairs

## Abstract

This paper proposes a mode-locked fiber laser based on graphene-coated microfiber. The total length of the fiber laser resonant cavity is 31.34 m. Under the condition of stable output of bright-dark soliton pairs from the fiber laser, dual-wavelength tuning is realized by adjusting the polarization controller (PC), and the wavelength tuning range is 11 nm. Furthermore, the effects of polarization states on bright-dark solitons are studied. It is demonstrated that the mode-locking state can be switched between conventional solitons and bright-dark solitons in the graphene mode-locked fiber laser. Bright-dark soliton pairs with different shapes and nanosecond pulse width can be obtained by adjusting the PC and pump power.

**Graphical Abstract:**

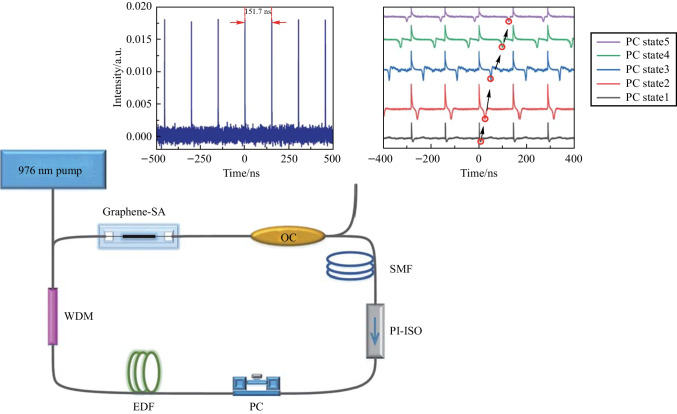

## Introduction

Passive mode-locking technology uses a saturable absorber (SA) in a resonant cavity to generate ultrashort pulses. Such a system can be realized by an all-optical fiber structure, and thus has the advantage of low cost. Artificial SAs including nonlinear loop mirror (NOLM), nonlinear amplifying loop mirror (NALM), and nonlinear polarization rotator (NPR) [1–3] have poor environmental stability. While natural saturable absorbers mainly include single-walled carbon nanotube (SWCNT), semiconductor saturable absorber mirror (SESAM) [4, 5], and graphene. The manufacturing and packaging of SESAM is complex and costly; the damage threshold of SWCNT is relatively lower, which affects the stability of laser pulse.

Graphene has a unique energy bandgap structure, a low absorption coefficient, a large modulation depth, and an ultrawide operating spectral range (300–2500 nm). In 2009, Bao et al. reported, for the first time, graphene-based passive mode-locked fiber laser generating conventional soliton pulses (pulse width 1.17 ps, 3 dB bandwidth 5 nm) [6]. Researchers have succeeded in many graphene mode-locking experiments and reported various types of output pulses. In 2010, Zhang et al. reported that a graphene fiber laser produced dissipative soliton pulse output in the positive dispersion region (pulse width 49 ps, 3 dB bandwidth 7.2 nm) [7]. In 2015, Sotor et al. used graphene as a SA with dispersion compensation in the cavity to produce dispersion-managed solitons (pulse width 88 fs) [8]. In 2021, Tang et al. reported that a graphene fiber laser generated noise-like pulses (pulse width 650 fs) [9]. All of the above-reported scheme generated bright solitons, however, according to the energy distribution law, laser pulses can be divided into bright and dark pulses. The bright-dark soliton pairs are the fixed solutions to the initial boundary value problem of the nonlinear Schrödinger equation (NLSE) [10]. The cross-phase modulation (XPM) effect generated by nonlinear transmission in optical fibers allows bright-dark soliton pairs to be interdependent. In 2013, Zhao et al. obtained bright solitons (pulse width 0.7 ps) and dark solitons (pulse width 8.7 ns) based on a graphene-oxide mode-locked fiber laser [11]. In 2014, Gao et al. obtained bright-dark pulse pairs (3 dB bandwidth 1 nm) using a graphene SA [12]. In 2018, Markom et al. built a zirconia-based erbium-doped fiber laser with a graphene oxide SA, which could generate bright solitons (pulse width 0.6 ps) and dark solitons (pulse width 20 ns) [13]. However, the graphene mode-locking devices used in these experiment were transmission-type and introduced significant insertion loss in the cavity. Coating graphene on a microfiber can solve the problems of low modulation depth and high loss with the transmission-type mode-locking devices. When the laser propagates in such a microfiber, the light propagating in the core has a strong birefringence effect due to the asymmetric structure of the microfiber.

In this paper, two switchable types of solitons are generated in the mode-locked state of a graphene fiber laser. First, the conventional mode-locked solitons are generated at 1558.56 nm, with pulse duration of 691 fs. By increasing the pump input power and rotating a polarization controller (PC) in the laser cavity, the conventional soliton mode-locked state is converted to the mode-locked state with bright-dark soliton pairs. The fiber laser outputs dual-wavelength pulses at 1560.5 and 1562 nm with a pulse sequence of 151 ns and a signal-to-noise ratio (SNR) greater than 52 dB. The output characteristics of two types of switchable mode-locked pulses, the evolution process of the bright-dark soliton pairs, and the dual-wavelength tunable characteristics are studied, for the graphene mode-locked fiber laser. The study will allow further broadening of the application of graphene in fiber lasers.

## Graphene SA preparation and modulation depth measurement

**Fig. 1:**
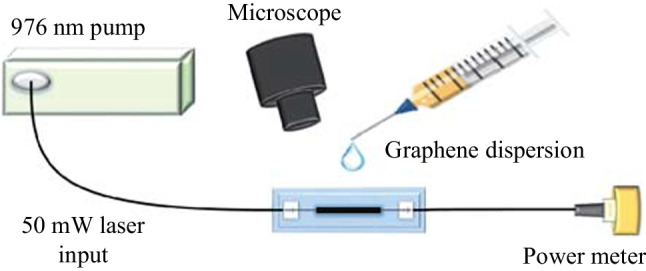
Optical deposition method for preparing graphene SA

The method to prepare graphene in the experiment is shown in Fig. 1. The first step is to disperse 2 mL of monolayer graphene solution into 50 mL of ethanol and then conduct ultrasonic treatment and centrifugation for about 5 h to obtain graphene dispersion. A 976 nm semiconductor laser source as a pump is connected to a microfiber. A power meter detects the power change after the light passes through the microfiber. Then, the graphene dispersion is dropped onto the tapered waist region of the microfiber. When the 50 mW pump light passes through the microfiber, a strong evanescent field generates at the tapered waist region, and graphene is rapidly deposited on the surface of the microfiber.

**Fig. 2:**
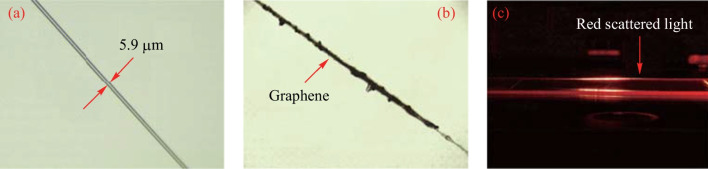
Images of graphene deposited microfiber. **a** Image of the microfiber. **b** Image of microfiber surface-coated with grapheme. **c** Scattering of red light in the microfiber

Figure 2a shows a microfiber with a tapered waist diameter of 5.9 μm prepared by the melt-drawn method. The image of graphene-coated microfibers is shown in Fig. 2b, and the graphene deposition length is 122 μm. After the graphene is deposited, the insertion loss of the microfiber increases from 0.5 to 3 dB. As shown in Fig. 2c, a strong scattering of visible light occurs when the visible red light passes through the microfiber.

**Fig. 3:**
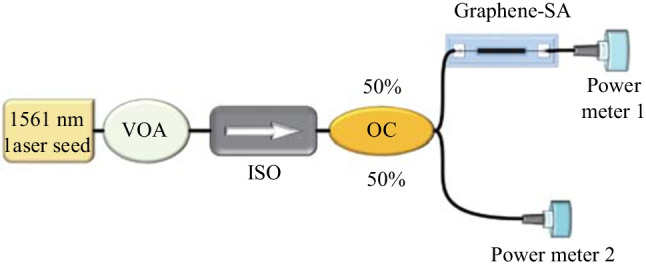
Double arm detection method for measuring the modulation depth of the graphene SA

**Fig. 4:**
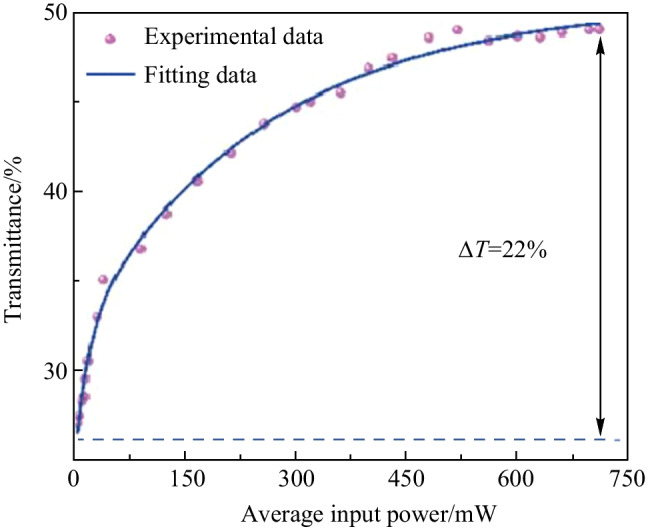
Transmittance of graphene with increased optical power, showing graphene modulation depth of 22%

As shown in Fig. 3, the double-arm detection method is used to measure the modulation depth of the graphene-saturated absorber. A ring cavity laser with a central wavelength of 1561 nm and a pulse width of 993 fs is built as the seed source. The pump light intensity was tuned through a variable optical attenuator (VOA). An isolator (ISO) is used to prevent feedback light from entering into the seed source. A 50:50 optical coupler (OC) is connected behind the isolator so that the laser pulse is transmitted in two optical paths, one for measuring the light power after passing through the graphene and the other for directly measuring the light power as a reference. Figure 4 shows the experimental data with the corresponding nonlinear fitting curve. Modulation depth of the graphene SA is found to be 22%, and the graphene reaches full saturation when the pump power increases to 750 mW.

## Experimental setup

**Fig. 5:**
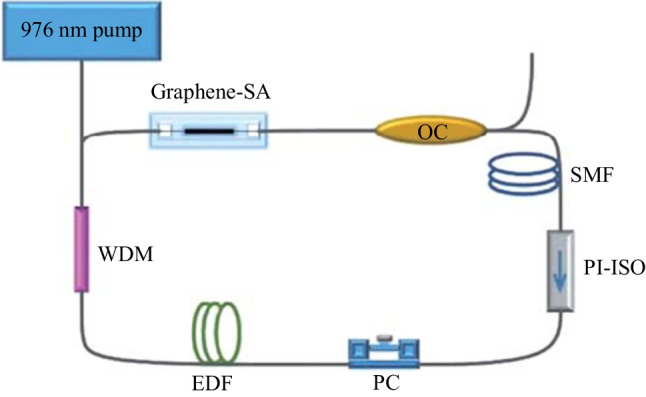
Image of the graphene-based laser resonator

Using the graphene SA prepared by coating graphene on a microfiber, a mode-locked fiber laser is built, and the experimental setup is shown in Fig. 5. The total length of the resonant cavity of the laser is 31.34 m, and the erbium-doped fiber (EDF) has a length of 5.5 m. The dispersion coefficient of EDF is 20 ps/(nm·km), and that of the single-mode fiber (SMF) is − 23 ps/(nm·km), the net dispersion is − 0.5027 ps^2^. The pump light enters the EDF through a wavelength division multiplexing coupler (WDM). Due to the graphene in the cavity, the cavity is polarization-dependent. The polarization state can be adjusted by a PC, and unidirectional propagation in the laser cavity is ensured by a polarization-independent isolator (PI-ISO). The SMF is connected to the 80% port of an OC, and the 20% port serves as the output of the resonant cavity. The test apparatus used in the experiment are: an optical spectrum analyzer mainly (Yokogawa, AQ6357) measures the output spectrum of laser output; a 1.5 μm band InGaAs photodetector (KG-PD-50G-DC-FA) converts optical signal to electrical signal; the time domain pulse is then measured with an oscilloscope (Agilent, DSO9254A), and the RF (Radio Frequency) spectrum of the pulse is measured by a spectrum analyzer (Agilent, N1996A) with the maximum measurable spectral range of 2.5 GHz. As the oscilloscope cannot measure a pulse duration of less than 90 ps, so it is necessary to use an autocorrelator (FR-103XL) to measure the actual pulse width.

## Results and discussion

### Generation of conventional solitons

**Fig. 6:**
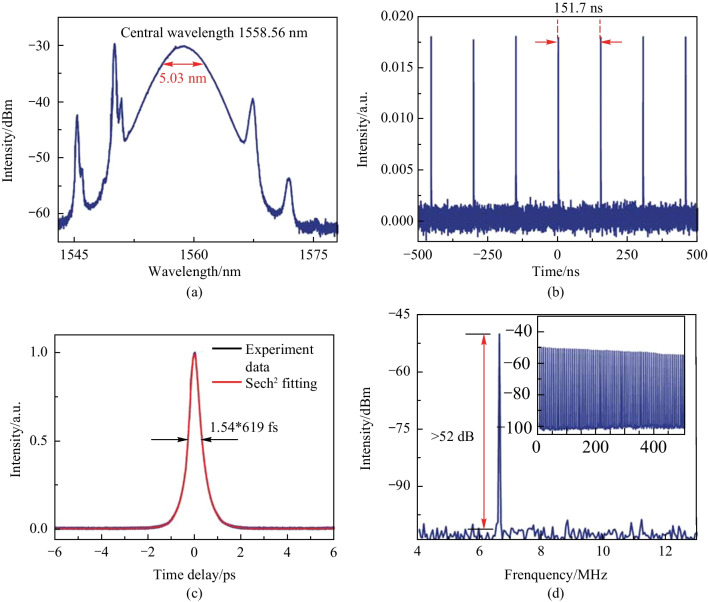
Output characteristics of conventional solitons. **a** Spectrogram. **b** Pulse sequence of conventional solitons. **c** The autocorrelation trace. **d** The RF spectrum. Inset: The RF spectrum in the 500 MHz range

When the pump input power is increased to 400 mW, and the polarization state in the cavity is appropriately changed by the PC, the stable conventional soliton state is observed. Figure 6a shows that the laser output spectrum has a clear Kelley sideband with a central wavelength of 1558.56 nm and a 3 dB bandwidth of 5.12 nm. The output power of laser is 4.4 mW. Through calculation formula for single pulse energy $E=\frac{P}{f}$ (where *E* is the single pulse energy, *P* is average output power, and *f* is fundamental repetition rate), the pulse energy is determined to be 0.667 nJ. The conventional soliton pulse sequence obtained is shown in Fig. 6b. The time interval between two adjacent pulses is 151.7 ns. The autocorrelation trace of the femtosecond pulse in the conventional soliton state is shown in Fig. 6c. The FWHM of the autocorrelation trace is 953 fs, and its actual pulse width is 619 fs. Τhe time-bandwidth product $\text{TBP}=\tau \cdot c\cdot \frac{\Delta \lambda }{{\lambda }_{c}^{2}}$ (where *τ* is the pulse duration, *c* is the speed of light in vacuum, $\Delta \lambda$ is the 3 dB bandwidth, and ${\lambda }_{c}$ is the center wavelength), is the product of the time-domain width of the output pulse and the 3 dB spectral bandwidth. The TBP is calculated to be 0.391, indicating a small chirp in the pulse. Figure 6d shows that the RF spectrum has an SNR exceed 52 dB, indicating that the laser is operating in a stable mode-locked state. The fundamental frequency is 6.6 MHz. The inset of Fig. 6d displays the broadband RF spectrum across 500 MHz range. The fundamental repetition rate of the pulse train can be calculated by $f=c/(nL)$ [14], where $c$ is the speed of light, $n$ is the refractive index of the fiber core, and *L* represents the length of the resonant cavity. When the cavity length is 31.34 m, the calculated fundamental frequency is 6.6 MHz.

### Generation of bright-dark solitons

**Fig. 7:**
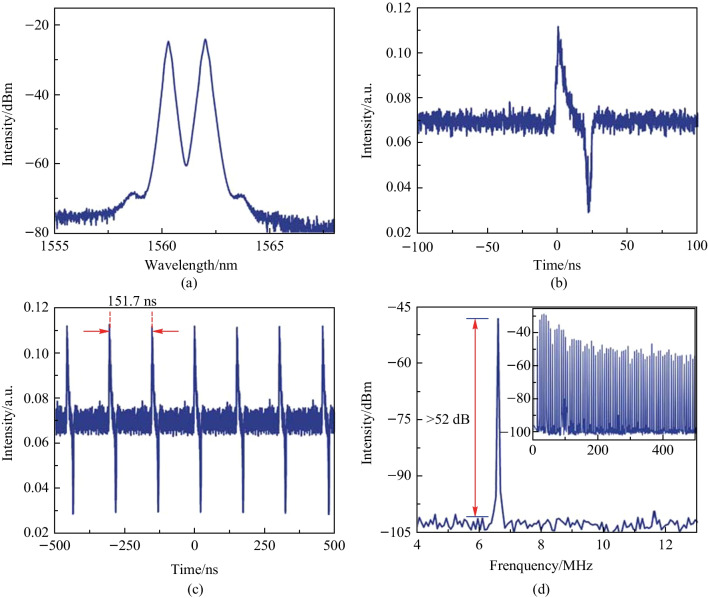
Bright-dark pulse output characteristics. **a** Spectrogram. **b** A bright-dark pulse. **c** Pulse train. **d** The RF spectrum. Inset: The RF spectrum in the 500 MHz range

By increasing the pump power to 650 mW and properly adjusting the polarization state in the cavity, the fiber laser can operate in a dual-wavelength mode-locked state. The dual-wavelengths are located at 1560.2 and 1562 nm, as shown in Fig. 7a. The separation between the two wavelengths is 1.8 nm. Graphene is highly nonlinear, as a result, four-wave mixing (FWM) occurs when light passes through graphene [15]. The uniform broadening of erbium-doped fibers means that each erbium ion radiation at the high-energy level has the same spectral line broadening effect. FWM in the graphene can suppress mode competition in the laser cavity caused by the homogenous broadening of the optical gain of the EDF [16], as FWM leads to energy transfer between wavelengths. Through the dynamic balance between FWM-induced energy transfer and mode competition, multi-wavelength laser output can be expected. As the graphene SA only has a single layer of graphene that interact with light through evanescent field, the nonlinear effect of graphene in our experiment is not strong enough, energy conversion to the two weak idler wavelength cannot cancel the effect of mode competition, so the dual-wavelength lasing is observed in the experiment. In addition, the spectral filtering effect caused by the combination of birefringence effect caused by the microfiber and the single mode fiber in fiber lasers and polarization effect of PC also contributes to the generation of the dual-wavelength [23]. The bright-dark pulse pairs can be observed by adjusting the trigger level of the oscilloscope to be near the noise floor. The pulses above the noise floor are bright pulses, and the pulses below the noise floor are dark pulses. As seen in Fig. 7b, the waveform of one bright-dark soliton pair is observed with the oscilloscope. Figure 7c depicts a sequence of bright-dark solitons. Since the cavity length of the resonant cavity does not change, the time interval between two adjacent bright-dark pulses is the same as that of the conventional soliton pulses. As shown in Fig. 7d, the mode-locking fundamental frequency is 6.6 MHz, and the SNR exceeds 52 dB. The inset in Fig. 7d suggests that the bright-dark soliton state is very stable through the uniformity of the spacing between the two frequency lines is good in the 500 MHz broadband RF spectrum. In dual-wavelength mode-locking, pulses at two different central wavelengths have different group velocity dispersions. The dispersion effect in the fiber causes changes in the pulse width of optical pulses, resulting in small fluctuations in pulse intensity. Therefore, a small amount of spectral modulation is found in the broadband spectrum, as shown in the inset of Fig. 7d.

### Characterization of bright-dark soliton pairs

**Fig. 8:**
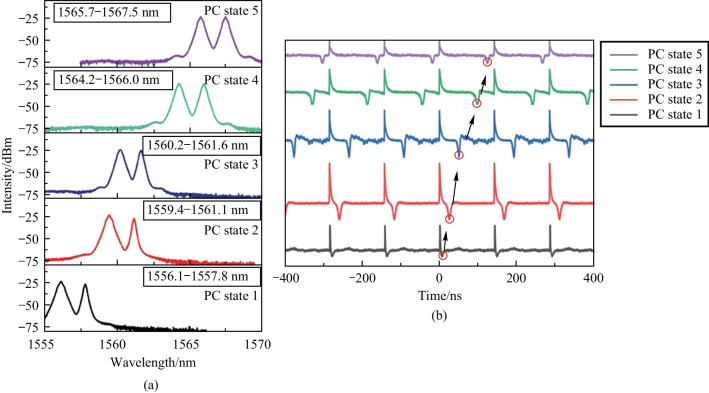
Experimental results of tunable dual-wavelengths by adjusting the PC. **a** Dual-wavelength tuning range. **b** Evolution of bright and dark solitons with different states of the PC

By adjusting the polarization states in the laser cavity, the dual-wavelength of the bright-dark solitons can be tuned, as shown in Fig. 8a, where the pump power is 700 mW. One peak of the dual-wavelength is tuned from 1556.1 to 1565.7 nm and the other from 1557.8 to 1567.5 nm. The polarization dependence of the microfiber with graphene coating originates from different losses in the two electric field directions that are perpendicular and parallel to the graphene layer, leading to a significant polarization extinction ratio [17, 18]. The bright and dark soliton pulses are located at different wavelengths, where the shorter band corresponds to the dark pulse and the longer band corresponds to the bright pulse, which has also been reported in Ref. [19] and will be discussed later in detail. Adjusting the PC changes the intensity and relative time position of the two kinds of pulses [20], so the bright and dark pulses can compete with each other, resulting in changes of the shape of the bright-dark soliton pairs. However, the spectrum retains a dual-wavelength feature, with two wavelengths move simultaneously from the shorter wavelength to the longer wavelength range [21]. The wavelength tuning process does not sacrifice the characteristics of bright and dark soliton pairs output by the fiber laser. The polarization tuning characteristics of the bright-dark solitons are studied experimentally, with the polarization controller rotates from 0 degrees. As shown in Fig. 8b, in polarization state 1, the rotation angle of the PC is slight, the bright and dark solitons are entangled with each other, and the spacing in the time domain is minimal. In polarization state 2, the interaction between bright and dark soliton is weakened, producing a more pronounced bright-dark soliton pair. The results indicate that the change of polarization direction changes the time spacing between the bright solitons and the dark solitons. As the rotation angle of the PC increases, the bright and dark soliton pulses tend to have a larger separation, and the separation is very obvious in polarization state 4 [22]. As the polarization angle increases further to polarization state 5, the separation is the maximum, and the interaction is the weakest, which can be equivalent to the generation of independent bright and dark solitons. When the PC is continuously adjusted, the polarization direction returns to polarization state 1, again bright-dark solitons in the mutually entangled state can be generated.

**Fig. 9:**
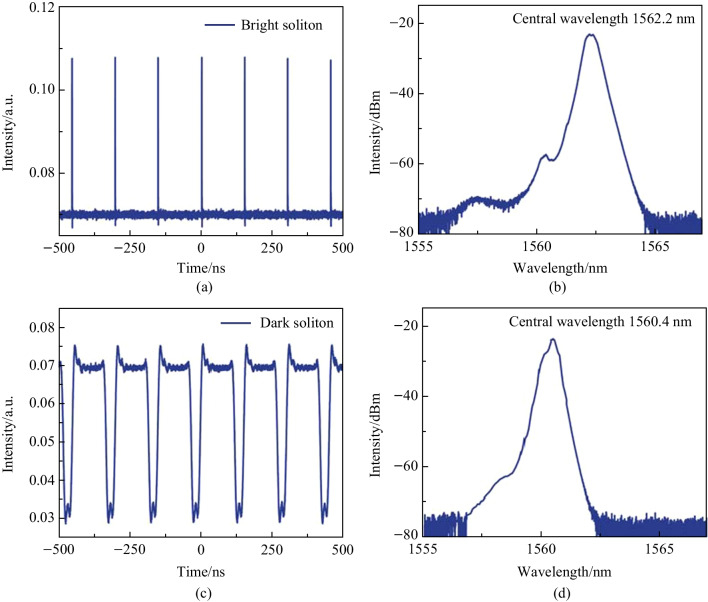
Separate measurement of bright-dark soliton pairs by PBS. **a** Bright soliton pulse sequence. **b** Corresponding spectrum of bright soliton pulse sequence. **c** Dark soliton pulse sequence. **d** Corresponding dark soliton pulse sequence spectrum

In the birefringent fiber laser, bright solitons and dark solitons propagate along the fiber with their electrical field directions parallel to the fast and slow axes of the fiber, respectively [12, 23]. The bright-dark soliton pair consists of two solitons with orthogonal polarization domain walls [24]. Both the birefringence effect of the microfiber and the weak birefringence effect of the SMF contribute to the birefringence effect of the fiber laser. By connecting a polarization beam splitter (PBS) at the output end of the fiber laser, the mode-locked bright and dark soliton pulses can be separated from the bright-dark soliton pairs after passing through the PBS, and the two kinds of pulses have orthogonal polarization directions. When the pump power is 650 mW, the results are as shown in Fig. 9a, one port of PBS outputs a bright soliton pulse sequence, and the corresponding spectrum is shown in Fig. 9b, with the center wavelength located at 1562.2 nm. As shown in Fig. 9c, the other port of PBS outputs a dark soliton pulse sequence, and the corresponding spectrum is shown in Fig. 9d, with the center wavelength located at 1560.4 nm. Thus, in the dual-wavelength optical spectrum of the bright-dark soliton pairs, the dark solitons correspond to the shorter wavelength band, while the bright solitons correspond to the longer wavelength band. Interaction between the two wavelengths eventually generates bright-dark soliton pairs in the negative dispersion region [19].

**Fig. 10:**
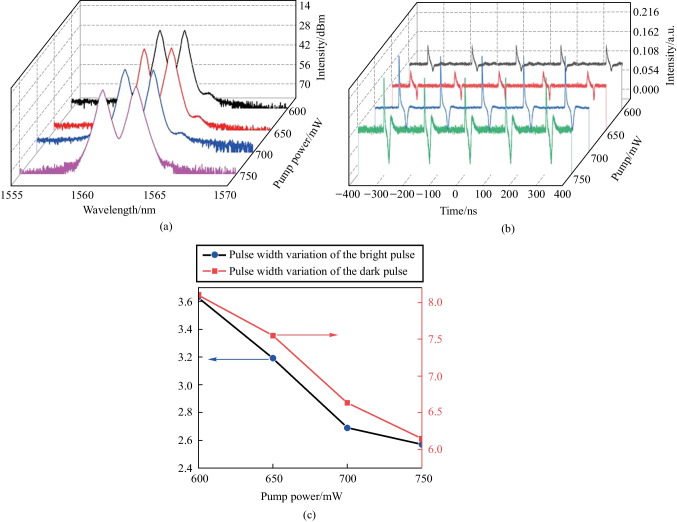
Experimental results showing the effect of the pump power: **a** Spectral distribution for different pump powers. **b** Bright-dark soliton pulses observed by the oscilloscope for different pump powers. **c** Pulse width change for bright solitons (dark solitons) with increasing pump power

The effect of pump power on the mode-locking state of bright and dark solitons is studied by increasing the pump power from 600 mW in steps of 50 mW. The spectra at different input powers are shown in Fig. 10a. When the pump power of the fiber laser gradually increases, and the spectrum is broadened, retaining the dual-wavelength feature, as observed on the spectrometer. Figure 10b shows the variation of the pulse sequence of the bright-dark soliton pairs as the input power increases. The intensity of bright solitons and dark solitons increases, and their pulse widths are compressed. As seen in Fig. 10c, as the input power increases, the pulse width of the dark soliton is compressed from 8.1 to 6.15 ns; that of the bright soliton is compressed from 3.63 to 2.57 ns. The results indicate that dark solitons have a faster compression speed.

**Fig. 11:**
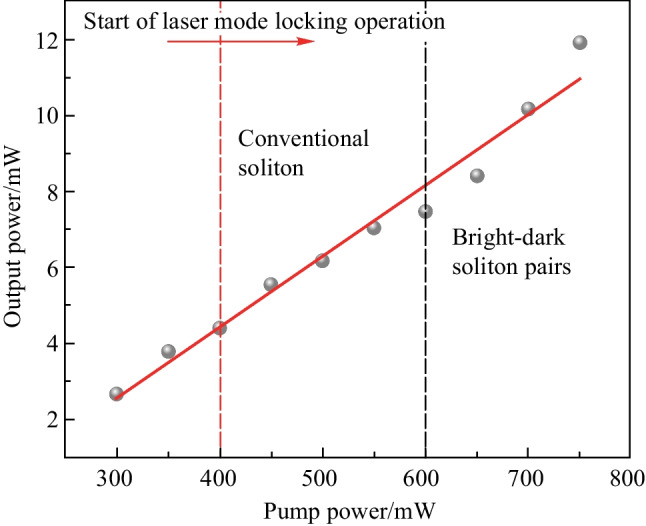
Output characteristics and two mode-locking states of the fiber laser

Figure 11 shows that the fiber laser has two modes of mode-locked state of operation for different pump powers: the conventional soliton state and the bright-dark soliton pair state. The optical–optical conversion efficiency is 1.6%. The mode-locking threshold of the fiber laser is 400 mW, and the conventional solitons are first observed by increasing the pump power from threshold level. When the pump power is 600 mW, the laser can output stable bright-dark soliton pairs. In the experiment, pulse compression of the bright and dark solitons is also found when the pump power is in the range of 600–750 mW. As the graphene reaches full saturation when the pump power increases to 750 mW, the pump power should be limited to less than 750 mW in order to obtain good mode-locking performance in the experiment and to avoid the possibility of graphene damage caused by excessive heat at high pump powers.

## Conclusions

In the mode-locked fiber laser based on graphene SA, both conventional solitons and tunable dual-wavelength bright-dark soliton pairs were observed. The conventional solitons have an actual pulse width of 619 fs and a fundamental frequency of 6.6 MHz. The conventional soliton mode-locked state can be transformed into bright-dark soliton state by changing the pump power. The tuning range of the dual-wavelength bright-dark solitons is 11 nm, which is realized by properly rotating the PC in the laser cavity. It is also found that bright and dark soliton pulses can be compressed by increasing the pump power. The conventional solitons with ultrashort pulse width and the dual-wavelength bright-dark soliton generated by the graphene mode-locked fiber laser may find various applications in different fields.

## Data Availability

The data that support the findings of this study are available from the corresponding author, upon reasonable request.
